# The Aurora-A inhibitor MLN8237 affects multiple mitotic processes and induces dose-dependent mitotic abnormalities and aneuploidy

**DOI:** 10.18632/oncotarget.2190

**Published:** 2014-07-09

**Authors:** Italia Anna Asteriti, Erica Di Cesare, Fabiola De Mattia, Volker Hilsenstein, Beate Neumann, Enrico Cundari, Patrizia Lavia, Giulia Guarguaglini

**Affiliations:** ^1^ Institute of Biology, Molecular Medicine and Nanobiotechnology (formerly Institute of Molecular Biology and Pathology), CNR National Research Council, c/o Department of Biology and Biotechnology, Sapienza University of Rome, Rome, Italy; ^2^ Advanced Light Microscopy Facility, EMBL, Heidelberg, Germany

**Keywords:** Aurora kinases, MLN8237, mitotic spindle, aneuploidy, time-lapse microscopy

## Abstract

Inhibition of Aurora kinase activity by small molecules is being actively investigated as a potential anti-cancer strategy. A successful therapeutic use of Aurora inhibitors relies on a comprehensive understanding of the effects of inactivating Aurora kinases on cell division, a challenging aim given the pleiotropic roles of those kinases during mitosis. Here we have used the Aurora-A inhibitor MLN8237, currently under phase-I/III clinical trials, in dose-response assays in U2OS human cancer cells synchronously proceeding towards mitosis. By following the behaviour and fate of single Aurora-inhibited cells in mitosis by live microscopy, we show that MLN8237 treatment affects multiple processes that are differentially sensitive to the loss of Aurora-A function. A role of Aurora-A in controlling the orientation of cell division emerges. MLN8237 treatment, even in high doses, fails to induce efficient elimination of dividing cells, or of their progeny, while inducing significant aneuploidy in daughter cells. The results of single-cell analyses show a complex cellular response to MLN8237 and evidence that its effects are strongly dose-dependent: these issues deserve consideration in the light of the design of strategies to kill cancer cells via inhibition of Aurora kinases.

## INTRODUCTION

The Aurora-A kinase is a major regulator of cell division and operates in distinct processes required for spindle assembly: in human cells it regulates separation and maturation of centrosomes at mitotic entry, mitotic microtubule (MT) nucleation [1- 3] and the integrity of spindle poles [2, 4, 5]. Recent data also indicate a role of Aurora-A in central spindle assembly at telophase [6, 7]. The highly homologous Aurora-B kinase also operates in control of the fidelity of chromosome segregation, by regulating chromosome condensation, correction of improper attachments between MTs and kinetochores, spindle checkpoint function, cytokinesis and abscission [8].

As other mitotic regulators, Aurora kinases are often abnormally expressed in tumor cells and are being investigated as targets of anti-mitotic compounds for cancer therapy [9, 10]. Many efforts have converged in the last years to develop Aurora inhibitors: molecules acting as ATP-competitors have been identified and some of them are currently in clinical trials [11]. Only a few of those molecules discriminate Aurora-A *vs* Aurora-B and may thus prove useful both in clinical studies for comparing the efficacy of anti-tumor responses and for dissecting the functions of Aurora kinases in mammalian cells.

MLN8237 (Alisertib) is a second generation Aurora inhibitor currently undergoing Phase-I/III clinical trials [11-16; www.clinicaltrials.gov]. Thus far, it is one of the molecules displaying highest specificity for Aurora-A over Aurora-B (300-fold in *in vitro* assays and 200-fold in HCT116 colorectal carcinoma cells [17]). Most pre-clinical studies based on whole cell population analyses in tumor cell lines showed cell growth inhibition, accumulation of polyploid cells over time, as well as induction of cell death [17-19]. Anti-tumor activity was also demonstrated in xenograft mouse models [17, 20, 21].

Available data on MLN8237-treated cells were mostly obtained from asynchronous cultures analyzed in bulk populations. This approach reveals the predominant cellular behaviour after long exposure to Aurora-A inhibition (24 to 96 hours) but can miss out transient phenomena and so mask the unfolding of relevant processes. In addition, inhibition of as pleiotropic a kinase as is Aurora-A, yields multiple phenotypes over time, making it difficult to dissect distinct functional roles within a bulk population. Microscopy-based single cell analyses are proving of critical importance to visualize the array of possible cell responses to anti-mitotic drugs [22]. Here we have coupled high resolution microscopy and high-throughput analysis of single cells treated with increasing doses of Aurora-A inhibitor to investigate the possible fates of cells with inactive Aurora-A.

A protocol was set up for treating pre-synchronized cultures when they reach G2 and analyze progression through G2 and mitosis as soon as Aurora-A inhibition is achieved. Because MLN8237 induces spindle pole abnormalities [23], we assessed the occurrence of chromosome mis-segregation events and aneuploidy induction, which would represent undesirable effects of the treatment in anti-cancer therapy. Our results highlight a partial specificity of MLN8237 in the U2OS cell line, with multiple cellular responses in a dose-dependent manner. The single cell analysis enabled us to depict a fraction of cells with defective spindle orientation, a defect that was not appreciated in previous studies of Aurora-A inhibition in human cells. In addition, we find that low and high MLN8237 concentrations yield mild and massive aneuploidy, respectively, representing a tumor-inducing or a tumor-suppressing condition [24]. Collectively, these results draw attention to the variability and the nature of cellular responses to the loss of Aurora kinase function, which may represent potential caveats deserving consideration when designing and interpreting clinical trials.

## RESULTS

### MLN8237 displays dose-dependent target selectivity on Aurora kinases

Prior to analyzing mitotic division in cells with inhibited Aurora-A, we sought to precisely define the specificity of MLN8237 inhibition in dose-response assays. We used the U2OS osteosarcoma cell line for its ease of cytological analysis, which renders it especially suitable for high-resolution single-cell microscopy analysis, and employed in our previous studies of RNA interference-mediated Aurora-A inactivation [4, 5, 23].

**Figure 1:**
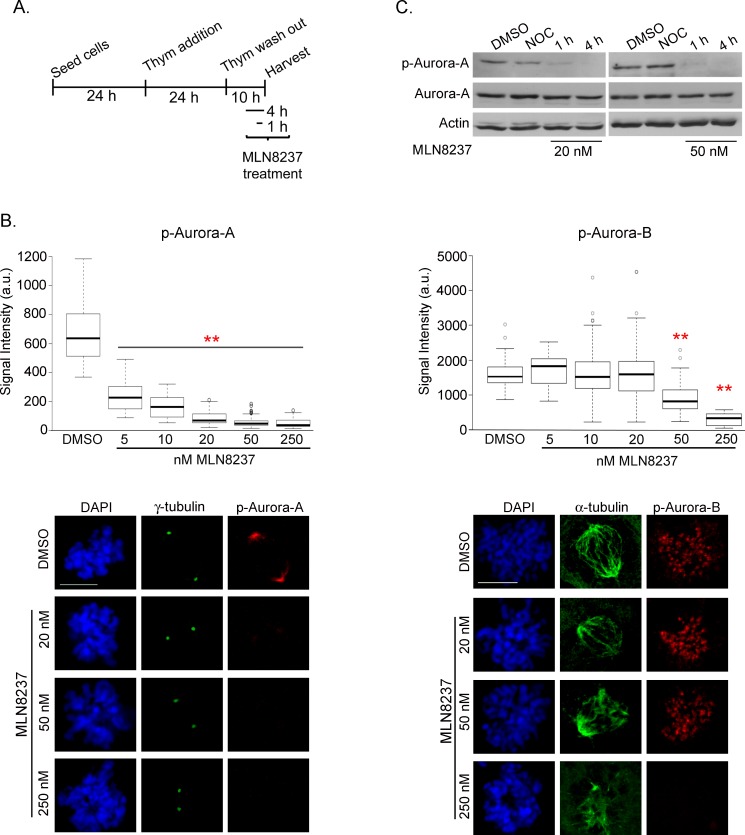
Dose-dependent inhibition of Aurora-A and Aurora-B by MLN8237 A. Protocol for MLN8237 treatment in cells progressing towards mitosis after thymidine (Thym) arrest and release. B. Quantification of IF signals for active pThr288-Aurora-A (left, mean intensity at poles) or active pThr232-Aurora-B (right, sum intensity at chromosomes) in control (DMSO) or MLN8237-treated prometaphases is shown in the box-plots (center lines show the medians; box limits indicate the 25th and 75th percentiles as determined by R software; whiskers extend 1.5 times the interquartile range from the 25th and 75th percentiles, outliers are represented by dots). Fluorescence intensity is shown in arbitrary units (a.u.). **: p<0.0001, unpaired t test or Mann-Whitney test. n=90 spindle poles (p-Aurora-A) or 50 prometaphases (p-Aurora-B) from 3 experiments. Representative IF images are shown. Scale bars: 10 μm. C. p-Aurora-A (active) levels decrease in mitotic extracts (shake-off) from MLN8237-treated (1 or 4 hours before harvesting) compared to DMSO- or nocodazole (NOC)-treated (4 hours) U2OS cultures. Total Aurora-A levels are also shown; actin is used as loading control. p-Aurora-B was not assessed in Western blot due to the lack of a suitable antibody for this application.

We set up a protocol by pre-synchronizing U2OS cells at the G1/S transition by thymidine treatment, then releasing from arrest into G2 and mitosis (Figure 1A). MLN8237 was added 6 hours after thymidine release (late S-phase/early G2) and cells were harvested after further 4 hours. Aurora-A activity was measured at the single cell level by anti-Aur-A-phospho-Thr288 immunofluorescence (IF) staining in dose-response assays (Figure 1B, left panels). Aurora-A auto-phosphorylation was significantly inhibited at concentrations ranging from 5 nM to 250 nM. With concentrations higher than 20 nM the residual signal at spindle poles was below 15% compared to controls. In Western blot analysis, no phospho-Thr288-Aurora-A was detectable in mitotic extracts from cultures treated with 20 and 50 nM MLN8237 for 4 hours, while some residual amount was present after 1 hour (Figure 1C).

Previous reports indicated that MLN8237 above 100 nM also inhibits Aurora-B activity in other cell lines [19, 25-27]. We therefore assessed the specificity of MLN8237 by measuring Aurora-B activity using anti-Aur-B-phospho-Thr232 antibody (Figure 1B, right panels). Surprisingly, we noticed that Aurora-B activity is already significantly compromised by 50 nM MLN8237; that was not evident when using anti-phospho-Histone-H3 (Ser10) as a reporter of Aurora-B activity (Supplementary Figure S1), possibly reflecting kinase redundancy or delay in detecting modulation of phosphorylation of downstream targets *vs* auto-phosphorylation.

Our single-cell analysis in U2OS cultures delimits therefore a narrow MLN8237 concentration window (20-50 nM) yielding effective and specific Aurora-A inhibition.

### MLN8237 delays mitotic entry and prolongs mitotic duration in a dose-dependent manner

**Figure 2:**
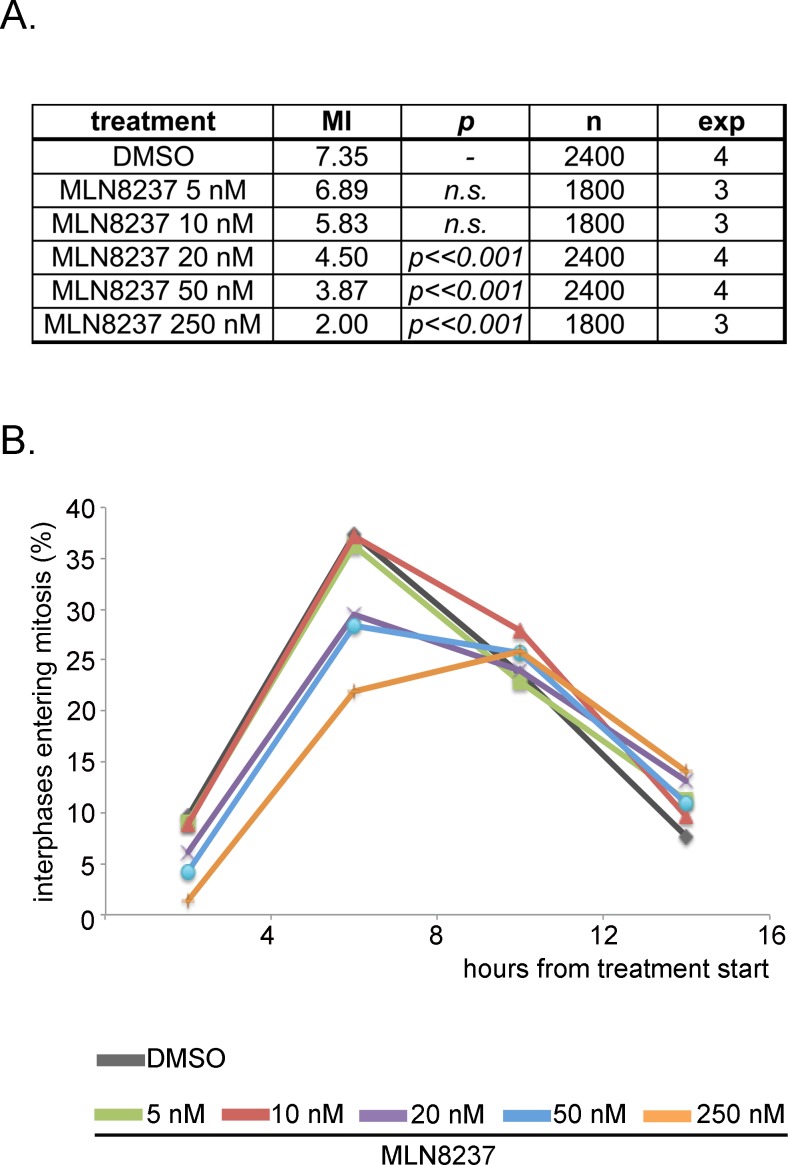
MLN8237 delays entry into mitosis A. Mitotic index (MI) from control (DMSO) and MLN8237-treated cultures (protocol as in Figure 1A), as assessed by DAPI (DNA) and alpha-tubulin (spindle) staining. p values (χ2 test) relative to control cultures, number of scored cells (n) and of independent experiments (exp) are indicated. B. Control (DMSO) and MLN8237-treated cultures were recorded by time-lapse imaging from the treatment start for the following 16 hours. The graph shows the percentage of interphases entering mitosis during the recording period; results are grouped in 4-hours intervals. 250 recorded interphases in 3 experiments for each condition.

We investigated the influence of MLN8237 on mitotic entry: after 4 hours of treatment, we found a significantly lower percentage of mitotic cells in MLN8237-treated cultures compared to controls (Figure 2A). This effect is dose-dependent, appearing at ≥ 20 nM, and is stronger at 250 nM MLN8237. To clarify whether cells were arrested in the G2 phase or rather delayed in progression through the G2/M transition, we recorded cultures in time-lapse experiments from the treatment start up to 16 hours later (Figure 2B). The peak of interphases entering mitosis in control cultures (DMSO) was between 4 and 8 hours from the treatment start (Figure 2B) and was not significantly affected by partial Aurora-A inhibition (5-10 nM MLN8237). Entry into mitosis was instead delayed above 20 nM MLN8237. In the 16 hours of time-lapse recording, 70-80% of the interphase cells entered mitosis in all treated cultures (about 60% with 250 nM MLN8237): thus, the majority of cells exposed to MLN8237 are delayed in G2, yet mitotic onset is not prevented.

**Figure 3:**
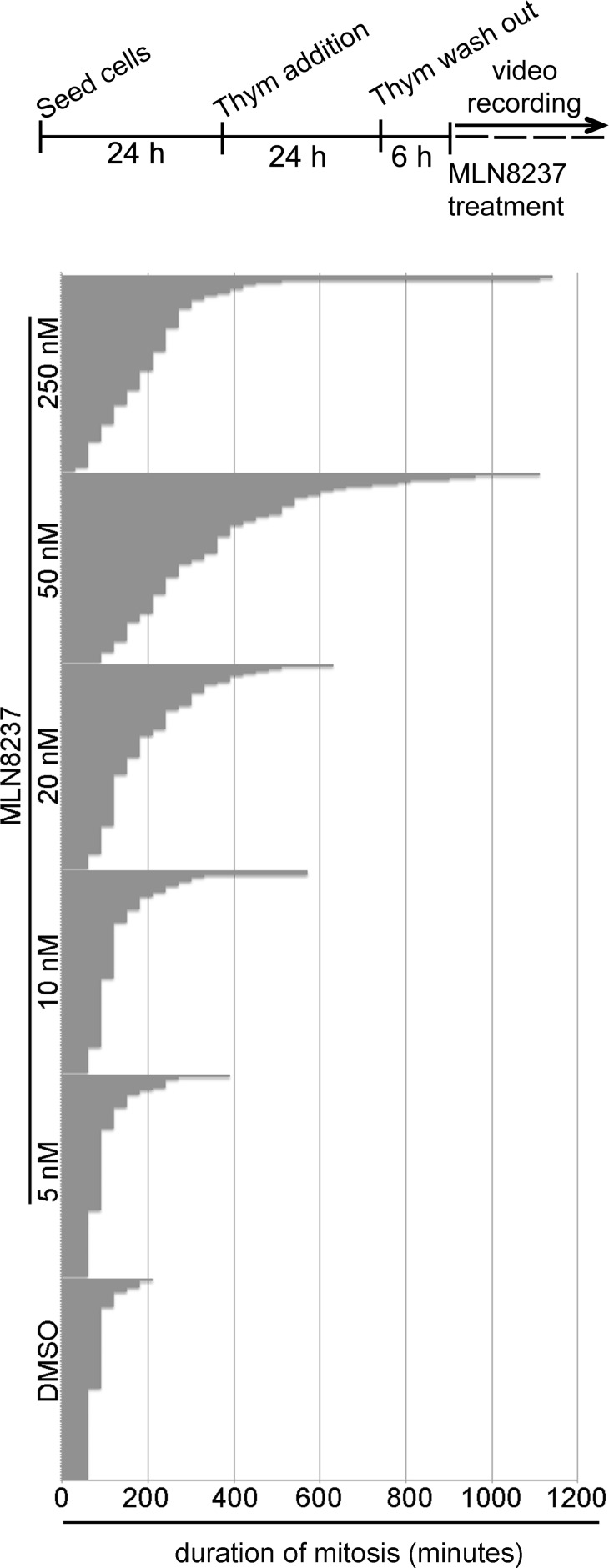
Dose-dependent lengthening of mitosis in MLN8237-treated cultures The protocol for time-lapse recording of MLN8237-treated cultures progressing towards mitosis from thymidine (Thym) arrest and release is depicted on top. Phase-contrast microscopy images were acquired with a 10x objective every 30 minutes. Duration of mitosis is calculated from round-up to visualization of 2 distinct daughter cells; each bar represents a mitotic cell. At least 90 cells per condition are displayed from 3 experiments.

Extending the time-lapse recording to 30 hours indicated that MLN8237 prolonged the duration of mitosis in a dose-dependent manner (about 300 minutes with 50 nM, compared to about 80 minutes in control cells; Figure 3). Importantly, although slowed down, cells eventually exited mitosis. Together, the results indicate that both the G2-to-mitosis transition and the overall duration of the mitotic process are strongly dependent on Aurora-A.

### Inhibition of Aurora kinases yields impaired MT nucleation, disorganized spindles and multipolar or failed cell division

We next analyzed spindle structure in cells that entered mitosis with different degrees of Aurora-A and Aurora-B inhibition.

**Figure 4:**
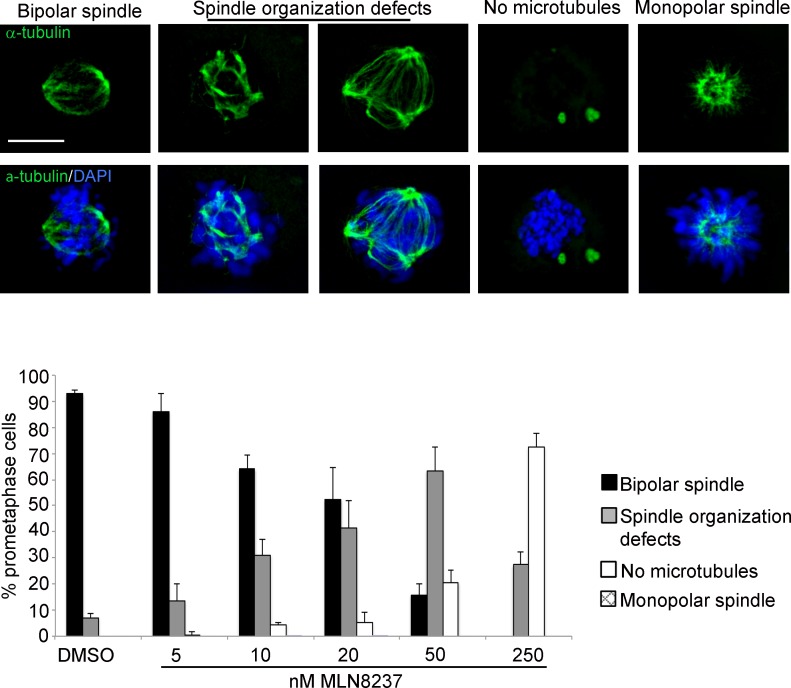
Spindle defects in MLN8237-treated mitoses Cultures harvested 4 hours after MLN8237 treatment (protocol as in Figure 1A) were stained for DNA and alpha-tubulin. Histograms represent the percentage of prometaphases displaying normal or defective spindles (IF panels on top). 250-300 counted cells per condition from 3 experiments; s.d. are shown. Scale bar: 10 μm.

With the highest MLN8237 concentration (250 nM) a strong impairment of MT nucleation was evident, with 70% of prometaphases displaying no MTs (Figure 4); this was associated with a prolonged prometaphase duration in time-lapse recording experiments, yielding an accumulation of prometaphase figures over all mitoses in fixed samples (Supplementary Figure S2). The MT nucleation defect was strongly dose-dependent and appeared in a relevant fraction of mitotic cells treated with 50 nM MLN8237 or above.

In cells in which MT nucleation was not visibly affected, spindles were highly disorganized (affecting 30% to 60% of all prometaphases with 10 nM or above; Figure 4); a fraction of these prometaphases displayed spindles with multiple poles, consistent with previous observations [17, 23]. A non-significant fraction of monopolar spindles was present at 10-20 nM MLN8237.

**Figure 5:**
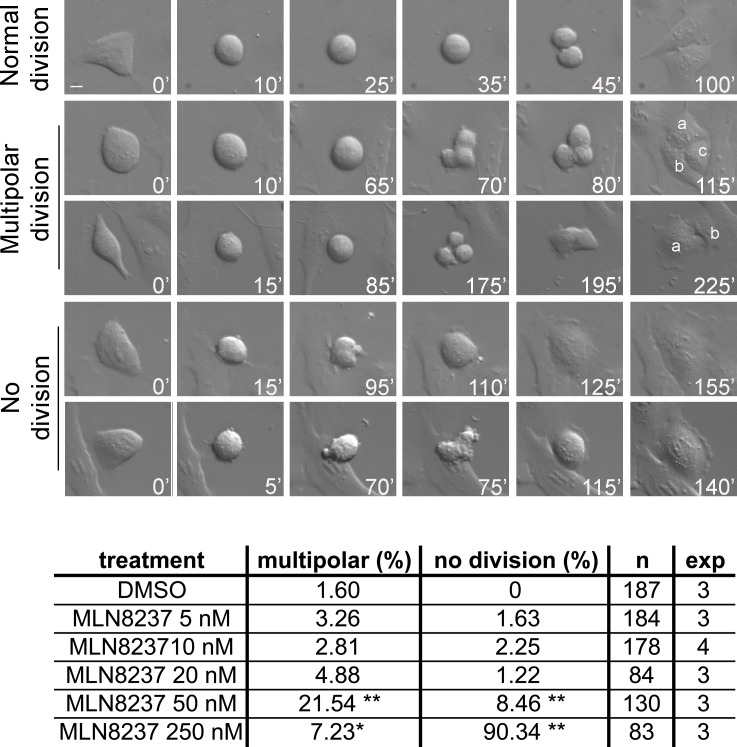
Time-lapse analysis of MLN8237-treated mitoses reveals multipolar and “no division” phenotypes Cultures treated as in the protocol in Figure 3 were recorded by time-lapse from treatment start for the following 24 hours. DIC images were acquired with a 40x objective every 5 minutes; representative single photograms are shown; time from round-up is indicated. First row: normal mitosis; second and third rows: multipolar mitoses (a, b and c indicate daughter cells); fourth and fifth rows: mitoses passing directly from prometaphase to defective interphase, with (lower) or without (upper) a “blebbing” phase. Defects are quantified (%) in the table below; number of recorded mitoses (n) and independent experiments per condition are indicated. *: 0.01<p<0.02; **: p< 0.001, χ2 test. Scale bar: 10 μm.

The influence of these defects on the global execution of mitosis was examined in depth in time-lapse movies of MLN8237-treated cells. Figure 5, first row, and Supplementary Movie S1 show examples of normal mitosis. We observed that a significant fraction of cells with inhibited Aurora-A (about 20% with 50 nM MLN8237, and a smaller fraction with 250 nM) underwent multipolar ana-telophase (Figure 5, second row; Supplementary Movie S2). Multipolar mitoses took a longer time to reach the stage of chromosome segregation (about 125 minutes average, compared to 35 minutes in control cells). In some cases two of the multiple groups of segregating chromosomes eventually re-joined, originating two asymmetric daughter cells (Figure 5, third row). Lower MLN8237 doses (10-20 nM), which yielded disorganized spindles in fixed prometaphases (Figure 4), do not yield multipolar divisions, suggesting that in those cultures a bipolar or pseudo-bipolar spindle is eventually assembled before anaphase.

In cultures treated with high MLN8237 doses we observed prolonged prometaphase (average duration 150-200 minutes) followed by a complete lack of chromosome segregation and cell division (Figure 5, fourth row): cells eventually re-adhered to form a single large or multinucleated interphase, often preceded by repeated “blebbing” movements (Figure 5, fifth row; Supplementary Movie S3). The “lack of division” phenotype appeared in a small fraction of mitoses treated with 50 nM MLN8237 and became predominant (about 90% of mitoses) with 250 nM. Similar phenotypes were previously observed in other human cell lines treated with high MLN8237 concentrations [26, 27]. Since the activity assays in Figure 1 indicate dual inhibition of both Aurora-A and -B under high doses, it was important to establish the contribution of each individual kinase to the no-division phenotype appearing above 50 nM. Small-scale time-lapse recording of cell cultures subjected to individual (Aurora-A or Aurora-B) or combined (Aurora-A+Aurora-B) RNA interference were set up to clarify this issue (Supplementary Figure S3): the no-division phenotype was not recorded in mitoses with selective inactivation of Aurora-A alone, yet appeared in a small fraction of Aurora-B defective cells (15,4%) and was amplified by the concomitant inactivation of both kinases (41,9%).

In summary, these results show that spindle organization is the most sensitive process affected by MLN8237 and is readily altered by even a partial reduction of Aurora-A activity, associated with unbalanced chromosome segregation. Stronger inhibition of the kinase induces impairment in MT nucleation, associated with prolonged prometaphase duration. The highest MLN8237 dose leads to a complete failure of cell division, largely ascribable to the concomitant inhibition of Aurora-B.

### Aurora-A inactivation induces defects in the orientation of cell division

**Figure 6:**
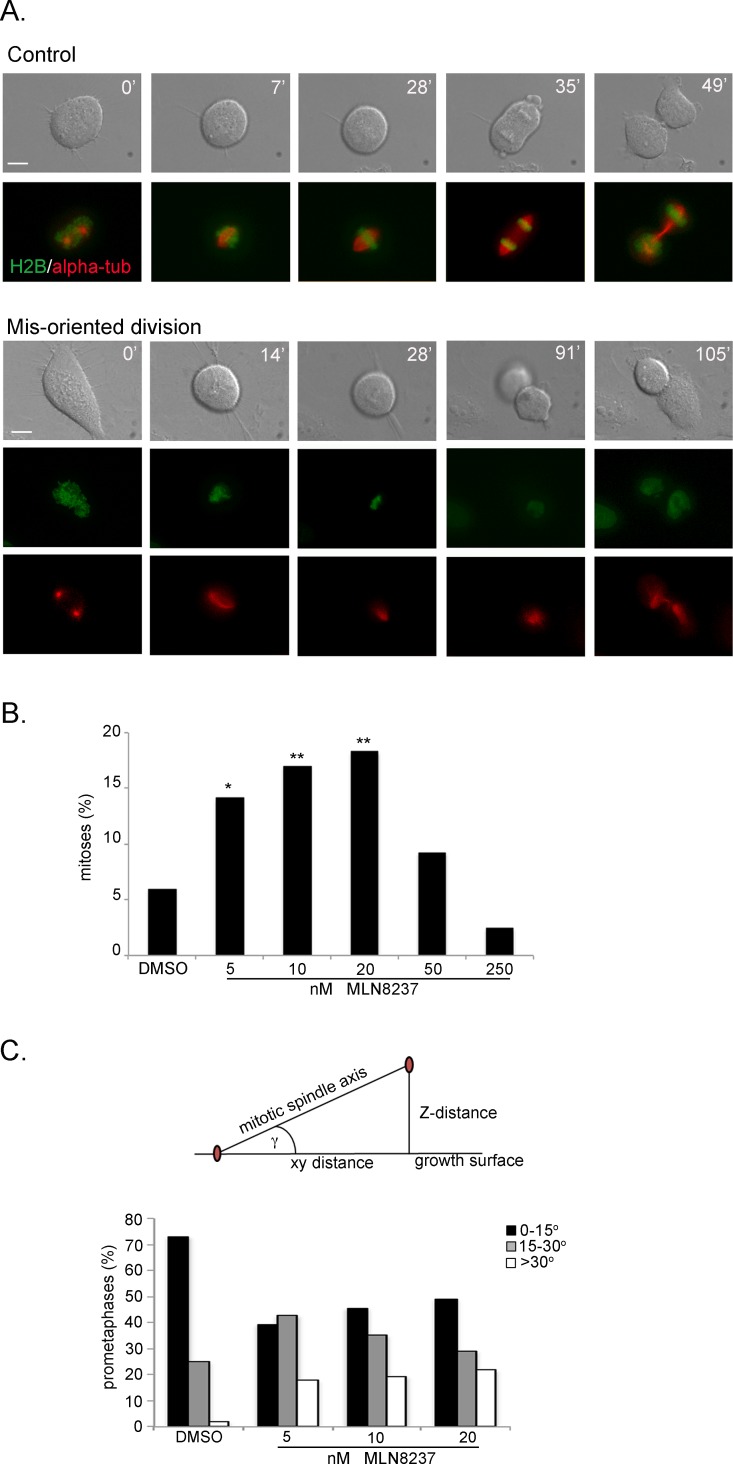
Aurora-A inhibition induces mis-oriented cell division A. An example of normally dividing cell is shown on top. Mis-oriented division in the presence of MLN8237 in U2OS cells with fluorescently labeled H2B (green) and alpha-tubulin (red) is shown below. Minutes from round-up are indicated. Scale bars: 10 μm. B. Quantification (%) of mis-oriented cell division recorded from MLN8237 treatment start for the following 20 hours (80-200 mitoses per condition from 3-4 independent experiments). *: 0.01<p<0.02; **: p< 0.01, χ^2^ test. C. The angle between the growth surface and the centrosome-centrosome axis in MLN8237-treated mitoses is calculated as schematized on top (centrosomes are in red; see Methods for details); histograms represent the distribution of prometaphases in 3 classes (50-60 cells from 3 independent experiments).

The time-lapse recording experiments also revealed that a fraction of cells did not divide parallel to the growing surface: that was already evident under conditions of partial Aurora-A inhibiton, occurring in about 15% of mitoses in U2OS cultures treated with 5, 10 or 20 nM MLN8237 (Figure 6A-B and Supplementary Movie S4). These cells took a longer time to reach the stage of chromosome segregation from mitotic round-up (on average 65 minutes with 5-10 nM and 110 with 20 nM MLN8237) compared to control cells (35 minutes). The time from the onset of chromosome segregation to re-formation of daughter interphase cells was instead unaltered, indicating that the process of chromosome segregation *per se* was not disrupted. However, we often recorded a delay between re-adhesion of the lower and the upper cell (see the example in Figure 6A and Supplementary Movie S4). No significant induction of mis-oriented division was observed above 50 nM MLN8237 (Figure 6B). In the case of 250 nM MLN8237 treatment, this is consistent with the predominance of the no-division phenotype (see Figure 5). The absence of mis-oriented divisions with 50 nM, which induced highly disorganized spindles and/or defective MT nucleation (see Figure 4), suggests that MTs are required and that spindle mis-orientation drives the abnormally oriented divisions.

To gain better resolution we used an U2OS cell line derivative stably expressing fluorescently labelled alpha-tubulin and histone H2B in video recoding assays (Supplementary Figure S4). MLN8237-treated mitoses that did not divide parallel to the culture dish displayed spindle rotation during prometaphase, such that often only one of the two poles was visible (Figure 6A, lower panels). To define the spindle orientation axis, we analyzed fixed samples and measured the angle formed between the centrosome-centrosome axis and the growing surface (Figure 6C). This analysis was performed in prometaphases from cultures treated with 5, 10 or 20 nM MLN8237 compared to controls. The average angle in control prometaphases was 11° and almost doubled (19°, mean value) in MLN8237-treated cells (p<0,01), reaching a >30° distortion in about 20% of prometaphases. Thus, the inhibition of Aurora-A under conditions that do not impair spindle formation altogether influences the proper orientation of the spindle axis and hence of cell division.

### Induction of aneuploidy in the progeny of MLN8237-treated mitoses

Some of the defects observed in MLN8237-treated mitoses suggest the possibility that genetically imbalanced daughter cells are generated.

**Figure 7:**
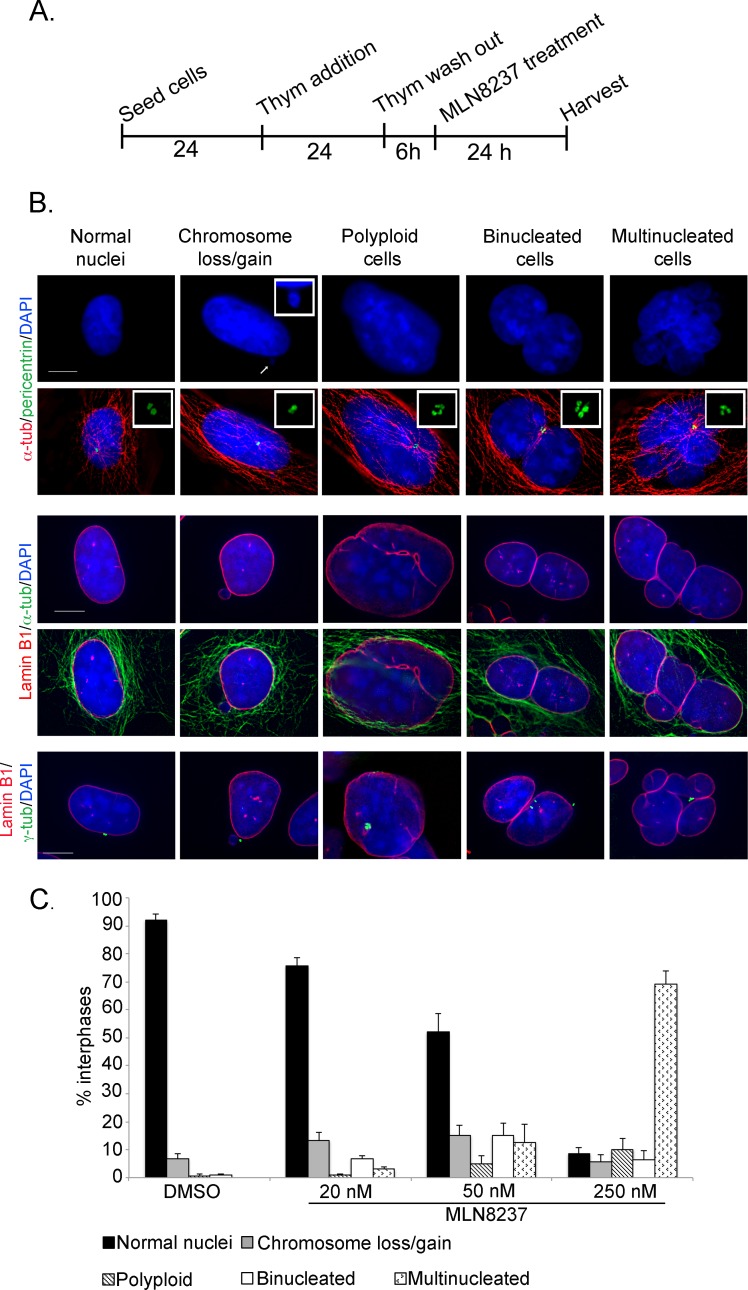
Outcome of mitoses treated with the MLN8237 inhibitor A. Schematization of the protocol for the analysis of the progeny of MLN8237-treated mitoses. B. The IF panels show different scored categories. Markers and color codes for DNA, MTs, nuclear envelope and centrosomes are indicated on the left. In the upper panels, the inset in the DAPI channel shows an enlargement of a micronucleus, while insets in the merged images show enlargements of centrosomes. Scale bars: 10 μm. C. Histograms represent the occurrence (%) of defects in B. At least 1000 cells per condition were counted in 3 experiments; s.d. are shown.

To address this issue we treated pre-synchronized cells with MLN8237 in G2 as described, fixed the cells after 24 hours and screened defects in interphase cells presumably representing the progeny of treated mitoses (Figure 7). Cells were stained with combinations of DAPI and antibodies against alpha-tubulin and pericentrin (examples in Figure 7B, upper panels) or lamin-B1 and alpha- or gamma-tubulin, middle and lower panels) and categorized: cells with loss or gain of 1 or few chromosomes (1-2 micronuclei), polyploid (1 large nucleus with 4 pericentrin or gamma-tubulin signals), binucleated, or multinucleated.

Indeed, following treatment with 250 nM MLN8237, most interphases appeared to have undergone chromosome mis-segregation (>65% multinucleated cells); a smaller fraction (about 10%) became polyploid. Only less than 10% of interphases were apparently normal (Figure 7C). Abnormalities were also observed in cells generated during treatment with 50 nM MLN8237: some 20% were multinucleated or polyploid and about 15% were binucleated. Interestingly, in about one third of the binucleated cells the nuclei were not equivalent in size, suggesting that they represent aberrant products of the multipolar/asymmetric divisions (see Figure 5). Time-lapse imaging of fluorescently labelled U2OS cells showed that some interphases remained very close after division with a connecting alpha-tubulin bridge (Supplementary Figure S4), possibly representing intermediate figures before binucleation.

**Figure 8:**
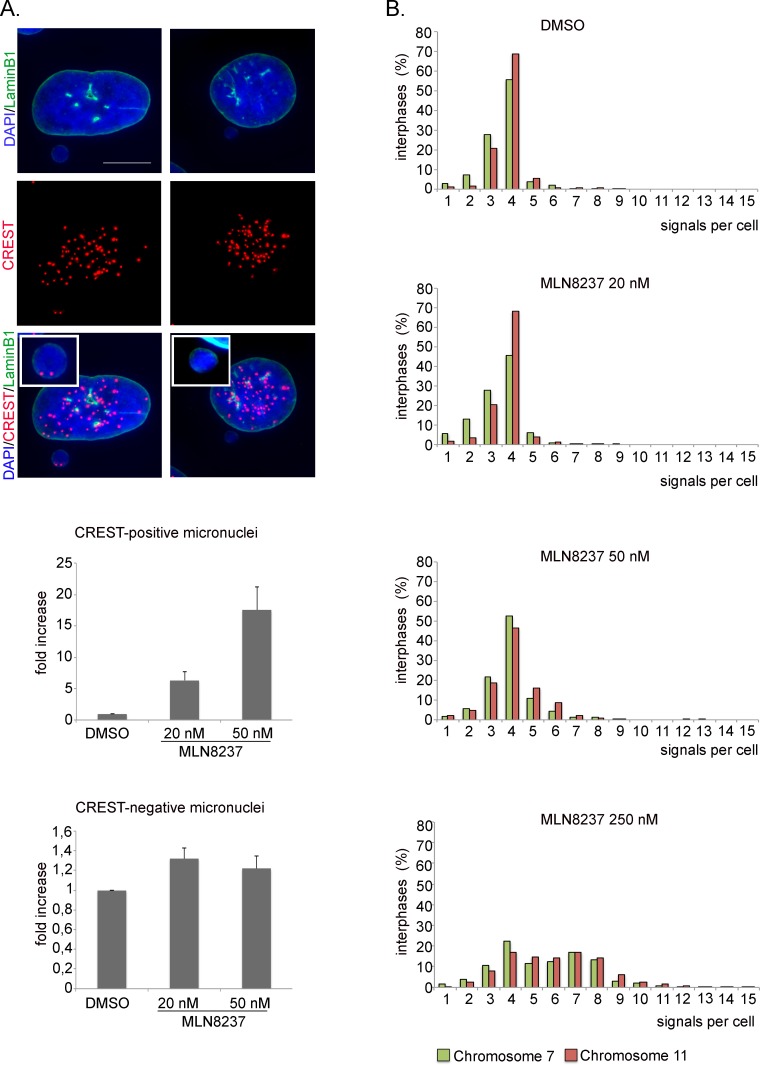
Ploidy alteration in MLN8237-treated cells A. IF images of CREST-positive (left panels) or -negative (right panels) micronuclei. The insets show enlargements of micronuclei. Histograms represent interphases with CREST-positive or -negative micronuclei in MLN8237-treated cultures (fold-increase relative to controls). 400 cells per condition were counted from 4 experiments. s.d. are shown. Scale bar: 10 μm. B. Distribution of interphases with different numbers of signals for chromosomes 7 and 11 (FISH hybridization). 800-1000 cells per condition were counted in 2 experiments.

20 nM and 50 nM MLN8237 also yielded a significant induction (14-15%) of cells with micronuclei, indicative of mild aneuploidy (Figure 7C). To assess whether micronuclei reflected genuine chromosome loss events, and hence aneuploidy, we assessed whether they contained whole chromosomes by staining with CREST antibodies to human centromeres (Figure 8A). We observed a 6- and 17-fold increase of CREST-positive micronuclei in 20 and 50 nM MLN8237-treated cultures, respectively, compared to controls. Consistent with this, recording of GFP-labelled chromosomes dynamically visualized chromosome bridges and micronuclei formation; interestingly, these defects were always present in mitoses with multipolar spindles (Supplementary Figure S4).

These results indicate different extents of ploidy alterations in MLN8237-treated cultures, which we decided to investigate directly by employing FISH (Fluorescence In Situ Hybridization) analysis. We counted hybridization signals produced by chromosome-specific centromeric probes (chromosome 7 and 11) in interphases originated from MLN8237-treated mitoses (protocol in Figure 7). Most (about 85%) control interphases displayed 3-4 signals for both chromosome 7 and 11 (Figure 8B), confirming that U2OS cells are hypertriploid. 20 nM MLN8237 induced a slight shift in the frequency of cells with fewer signals (2-fold increase in interphases with 1-2 signals compared to controls), likely corresponding to the chromosome loss events evidenced by the micronuclei analysis. With 50 nM MLN8237, cells displaying 3-4 signals decreased to about 70% and cells with > 4 signals correspondingly increased; a minor fraction of truly polyploid cells (≥6 signals for both chromosomes within a single large nuclei) was also identified, consistent with the IF results in Figure 7. With 250 nM MLN8237 > 50% interphases had ≥ 6 signals, indicative of gain of complete sets of chromosomes, consistent with the lack of division observed in time-lapse experiments (Figure 5) and with the frequent multinucleated state depicted in Figure 7. FACS analysis confirmed the increase in ploidy in about 45% of the viable population (data not shown) after 48 and 96 hours of treatment with 250 nM MLN8237.

Together these analyses indicate that MLN8237 treatment yields variable levels of aneuploidy in daughter cells, in a dose-dependent manner.

### Long term fate of MLN8237-treated cells

**Figure 9:**
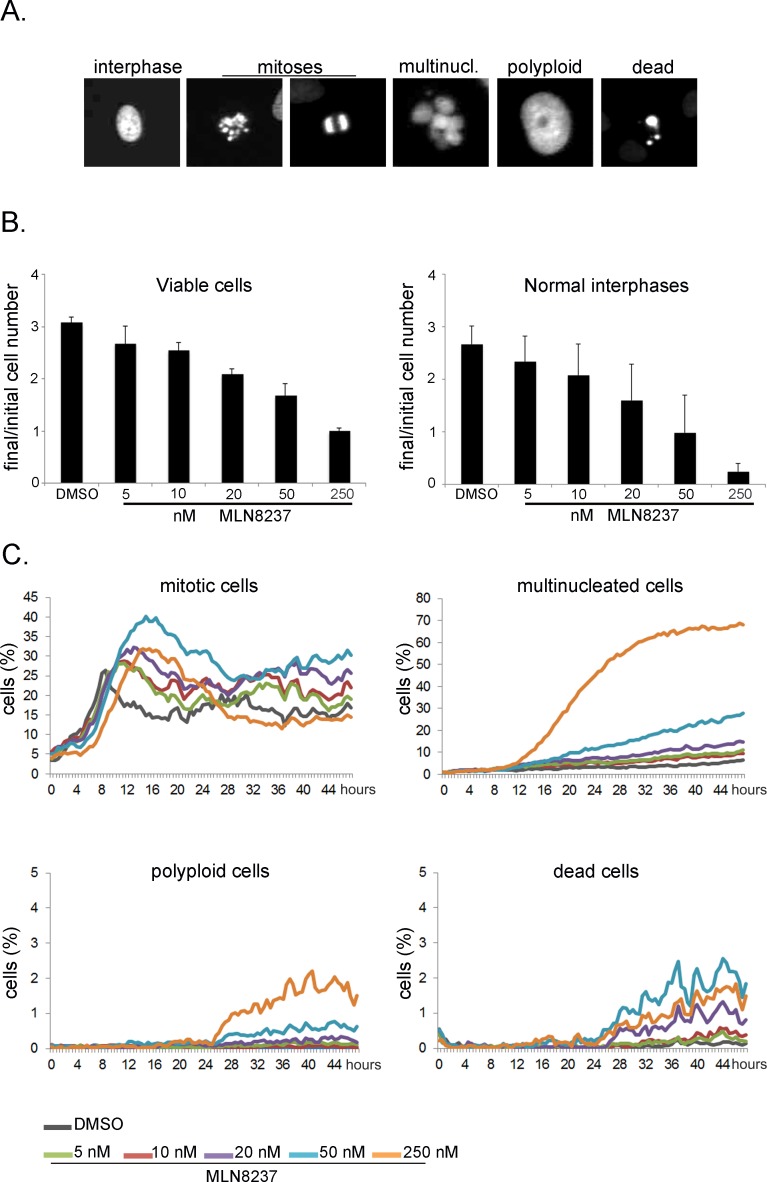
Long-term high-throughput analysis of MLN8237-treated cultures Cultures treated as in Fig. 3 were recorded for 48 hours. Automated segmentation and classification on the images was performed using the CellCognition software. A. Classes defined for training the classifier are shown with representative examples for each class. B. Histograms on the left represent the increase in the number of viable cells from the first to the last frame of the acquisition. Average values and s.d. of 10 replicates from 3 independent experiments are shown. The analyzed sample size at time 0 was at least 500 cells per replicate. Histograms on the right represent the increase in normal interphases only under the same conditions. C. The line charts represent the percentage of cells per class during the recording time.

The time-lapse analysis thus far indicates that MLN8237 (5-250 nM range) induces no significant cell death in U2OS mitotic cells. We investigated the long-term outcome of the treatment in high-throughput time-lapse analyses for a length of time (48 hours) roughly corresponding to 2 division cycles. Using the fluorescently labelled U2OS cell line, we analyzed the data with an automated method, the CellCognition software [28], trained to classify cells as interphasic, mitotic, dead, multinucleated or polyploid (examples in Figure 9A). In the 48 hours of the recording time, the cell number increased threefold in control cultures, yet dose-dependent growth inhibition was observed in cultures treated with MLN8237, with almost no increase with 250 nM MLN8237 (Figure 9B, left panel). Consistent with this, the number of normal interphase cells was dramatically reduced by the treatment (Figure 9B, right panel): this effect could result either from an increase in mitotic cells (due to prolonged mitotic duration) or from the generation of abnormal interphase cells. The kinetics of appearance of mitotic cells depicted two waves of division in control cultures (Figure 9C). In MLN8237-treated cultures the first mitotic peaks were shifted in time and appeared broader. Both effects were dose-dependent, consistent with our data on mitotic entry and duration (Figures 2 and 3). At 250 nM inhibitor, no second wave of division was observed. Concomitantly, we observed a strong increase of multinucleated cells at 250 nM and a milder effect at 50 nM MLN8237. Some polyploid cells appeared under these conditions (below 3%). Detection of multinucleated and polyploid cells was therefore consistent with the results from fixed samples (Figures 7 and 8). Importantly, the induction of cell death remained below 3% throughout the recording time (Figure 9C, bottom right panel), and remained at a similar level in one time-lapse experiment extended to 65 hours (not shown).

Together, the high-throughput data indicate that MLN8237 induces a dose-dependent lengthening of mitotic progression and the generation of abnormal daughter cells that are impaired in re-dividing, with a cytostatic effect, while low cytotoxicity is observed within the first 48 hours of treatment.

**Figure 10:**
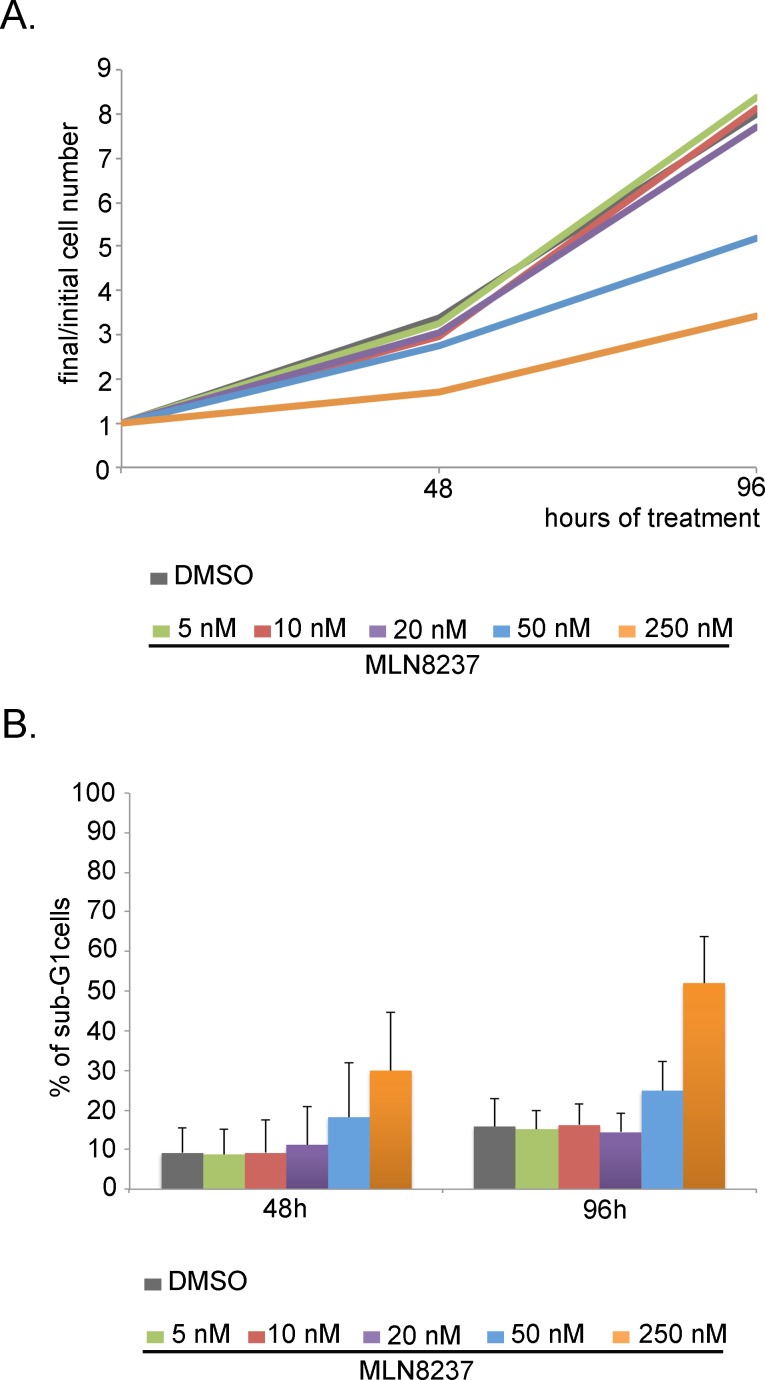
Dose-dependent effects of MLN8237 on cell growth and viability A. Cells were counted after 48 and 96 hours from MLN8237 treatment start. Values represent the increase in the number of cells respect to t=0h (3 independent experiments). B. The percentage of sub-G1 cells in MLN8237-treated cultures, detected by FACS analysis, is shown in the histograms (3 experiments). s.d. are shown.

We also performed proliferation/cell death analyses after several days (Figure 10) by cell counting and FACS measurement of the DNA content. After 48 hours of treatment, corresponding to the end of the high-throughput video-recording, a slight decrease in the total cell number was observed with 5-10-20 nM MLN8237 compared to control culters (Figure 10A); the overall proliferation trend remained comparable to controls over 96 hours. Cell growth inhibition was instead appreciated with 50 nM MLN8237 and even more effectively with 250 nM MLN8237 (Figure 10A).

To assess cell death we measured cells with a sub-G1 DNA content by FACS analysis (Figure 10B). That revealed a generally higher level of cell death compared to the microscopy analysis, possibly reflecting technical specificities in the methodology: indeed, detached dead cells are counted by cytofluorimetry, while being preferentially lost in microscopy analysis. Nevertheless, by FACS analysis, only 250 nM MLN8237 induced remarkable cell death (about 30% of sub-G1 cells after 48 hours of treatment, increasing to about 50% after 96 hours, compared to about 10% in control cultures; Figure 10B).

Thus, MLN8237 treatment in concentrations that genuinely inhibit Aurora-A does not efficiently promote cell death in U2OS cells; a toxic effects is only observed after very long exposure times to high doses that simultaneously target Aurora-B.

## DISCUSSION

Here we have used the MLN8237 small molecule inhibitor to investigate mitotic roles of the Aurora-A kinase in osteosarcoma U2OS cells; this molecule is under clinical trial in several cancer types including osteosarcoma.

We first determined the extent and specificity of kinase inhibition by assessing Aurora-A and Aurora-B self-phosphorylation. MLN8237 was effective over Aurora-A in the 20-50 nM range, but also inhibited Aurora-B above 50 nM. The loss of selectivity with high doses was previously reported [25-27]. The inhibition of Aurora-B identified here with 50 nM MLN8237 (a very close condition to that required for complete Aurora-A inhibition) was instead not noticed before using histone H3 phosphorylation as a reporter, indicating that the selection of activity reporters is critical to ascertain the selectivity of kinase inhibitors. It also evidences that the window at which MLN8237 fully inhibits Aurora-A, without concomitantly affecting Aurora-B, can be very narrow in some cell lines, indicating that selective inhibition of Aurora-A *vs* Aurora-B remains a critical issue, even with the best performing ATP-competitors.

MLN8237 treatment prolongs the G2 phase duration. The G2 delay was under-appreciated in previous studies using MLN8237 in asynchronous cultures, yet was observed when Aurora-A was inhibited by either antibody microinjection or RNA interference [1, 29], or by conditional ablation in mouse embryonic fibroblasts (MEFs; [30]). No permanent arrest is however induced, suggesting that Aurora-A functions in G2 are important, but can be taken over by other kinases. Indeed, the G2 delay is more severe with 250 nM, under which condition Aurora-B is also inhibited. These findings raise the possibility that centrosome- and/or MT-associated defects induced by Aurora-A inactivation in G2 evoke a checkpoint response that delays the transition towards mitosis onset.

**Figure 11:**
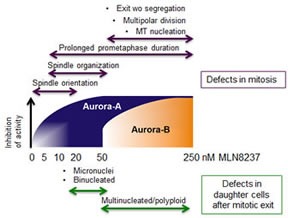
Dose-dependent effects of MLN8237 on cell division and aneuploidy induction: a schematic overview Schematic representation of the phenotype distribution (Up: mitotic defects. Low: abnormal daughter interphases) along a MLN8237 concentration gradient in U2OS cells. Gradual inhibition of Aurora-A (blue) and Aurora-B (orange) is indicated by the gradients within shapes (dose intervals are not in scale); note that at 20 nM MLN8237 Aurora-A inhibition is virtually complete.

Dose-response analyses of MLN8237-treated cells that entered mitosis revealed processes that are differentially sensitive to Aurora-A inactivation, as depicted in the schematics in Figure 11. Complete Aurora-A inhibition impaired MT nucleation; consistent with this, previous data showed that partial *vs* complete RNA interference-mediated depletion of Aurora-A differentially affects maturation of centrosomes, required for mitotic MT nucleation [1, 2]. Partial Aurora-A inhibition (10 nM MLN8237) instead yielded multipolar or disorganized spindles, the frequency of which increased in a dose-dependent manner up to 50 nM. Defective spindle assembly was associated with longer prometaphase duration compared to untreated cells. The highest frequency of induction of spindle organization defects (50 nM) was associated with multipolar divisions.

250 nM MLN8237 also prolonged prometaphase duration, after which cells re-adhered and eventually exited mitosis without division. The failure of chromosome segregation could be appreciated in time-lapse experiments and was consistent with previous results obtained from concomitant inactivation of Aurora-A and B kinases by high MLN8237 doses in Hep3B or HeLa cells or, to a lesser extent, following Aurora-B inhibition in HeLa cells [26, 27]. Our own recording experiments of interfered mitoses for Aurora-A, or -B, or both, confirm a mild effect of Aurora-B inactivation alone and a synergic effect of the inactivation of both kinases. Complementary functions of Aurora-A and B in chromosome segregation also emerged in chicken DT40 Aurora-A^KO^ cells treated with an Aurora-B specific inhibitor [31]. None of the approaches used to inactivate Aurora-A alone in human cultured cells yielded chromosome segregation failure [2, 27, 32]. The latter was instead described in Aurora-A-null MEFs [30, 33], suggesting specific modes of action of the two kinases in this cellular context, which may reflect a different stoichiometry between Aurora-A and B and/or their substrates.

An interesting finding from this study is the induction of mis-oriented divisions by low MLN8237 concentrations (5-20 nM); the inhibitor is selective for Aurora-A at these doses and, as recalled above, does not impair MT nucleation: this directly implicates the lack of Aurora-A in the mis-orientation phenotype and suggests that MTs are required. Interestingly, Aurora-A is implicated in spindle orientation in asymmetric cell divisions in Drosophila [34] via phosphorylation of Pins [35]. Excess Aurora-A can also influence the spindle orientation and the cell fate in human mammary epithelium stem/progenitor cells [36]. In our time-lapse assays using a U2OS cell line with fluorescent MTs, we actually visualized spindle rotation movements: thus, Aurora-A activity is required for pathways that determine the mitotic spindle orientation, raising the possibility that Aurora-A inhibition influences the fate of asymmetrically dividing cells and/or tissue architecture.

Time-lapse experiments also revealed that MLN8237 generates chromosome mis-segregation. At high concentrations, when both Aurora-A and Aurora-B are inhibited, massive aneuploidy is observed, with the generation of multinucleated daughter cells. Lower doses that inhibit Aurora-A alone yield mild aneuploidy, with increased number of CREST-positive micronuclei (schematics in Figure 11). FISH analysis depicted the differential generation of mild and massive aneuploidy by low and high MLN8237 doses, respectively. This is relevant, given the pro- or anti-tumorigenic effects of aneuploidy depending on the extent of chromosome mis-segregation [24]. Consistent with our observations, Yang and colleagues [37] described the generation of an 8N population following simultaneous RNA interference-mediated inactivation of Aurora-A and Aurora-B, but not of Aurora-A alone, in U2OS cells. Segregation defects (lagging chromosomes and chromatin bridges) were observed in Aurora-A-null MEFs, with increased ploidy over time [30, 33], again evidencing a crucial contribution of Aurora-A to chromosome segregation in this system.

We also observed binucleated cells in the progeny of MLN8237-treated mitoses; in time-lapse analyses, telophase cells often remained in closer proximity to one another compared to controls, and tubulin bridges persisted, a phenotype that may possibly evolve into a binucleated cell. The observation of binucleated cells with 20 nM MLN8237 would support a recently proposed direct role of Aurora-A in late steps of cell division [6, 7].

Importantly, MLN8237 failed to induce cell death in mitosis nor did it cause a highly effective elimination of the cellular offspring within the first 48 hours of treatment. Recent time-lapse studies with MLN8237 reported variable results regarding mitotic cell death: no mitotic toxicity was observed in Hep3B cells [26], whereas some death from mitosis was recorded in HeLa cells [27]. Aurora-A inactivation therefore appears to require a set of concomitant conditions, as yet elusive, for mitotic cell death activation. The highly aneuploid progeny generated in our assays at 50 nM (and, to a higher extent, 250 nM) MLN8237 originates cells impaired in further cell division, hence unviable in the long term. The mechanisms underlying the cytostatic effects of Aurora-A inactivation are controversial: a dose- and time-dependent induction of apoptosis was described in different cell lines treated with MLN8237 [18, 19, 38, 39], while in other cases the MLN8237-induced cytostatic effect is attributed to senescence [40] consistent with results described in Aurora-A-null MEFs [33], or to induction of differentiation pathways [41]. These observations suggest that both the treatment parameters and the cellular background contribute to determine the long-term outcome of MLN8237-treated cultures.

In conclusion, the broad variability in the U2OS cell response to MLN8237 highlighted in this study is an important issue in the light of the use of this compound in anti-cancer therapy. In the human organism undergoing treatment, the compound dose cannot be constant and is expected to rise and fall over time. It will be important to extend these studies and shed light on the pathways driving the response towards one or another cell fate, with the perspective to modulate such choice and drive cells towards death pathways.

## METHODS

### Cell cultures, synchronization protocols and treatments

The human U2OS osteosarcoma cell line (ATCC: HTB-96) was grown at 37°C in a 5% CO_2_ atmosphere in DMEM, supplemented with 10% faetal bovine serum. U2OS cells stably expressing H2B-GFP and RFP-alpha-tubulin (kind gift of L. Lanzetti, Institute for Cancer Research at Candiolo, Italy) were cultured under the same conditions and used in time-lapse imaging experiments. For synchronization, cells were subjected to a 24 hours block in 2 mM thymidine. Cultures were then released from the G1/S arrest by washing away the thymidine and adding fresh medium containing 30 μM deoxycytidine; 6 hours post-release cultures were treated with MLN8237 (5-250 nM as indicated; Selleck Chemicals) or 0,1 μg/ml nocodazole (Sigma Aldrich). Mock-treated cultures were incubated with DMSO.

### Cell counting and FACS analysis

Cells were harvested after 48 and 96 hours from treatment. One sample was harvested when cultures were released in thymidine–free medium (t=0h) as reference for the initial number of cells and for verifying the efficacy of the thymidine arrest. For counting the number of cells a Z1 Coulter Particle Counter (Beckman Coulter) was used. For FACS analysis samples were permeabilized with 0.1% TritonX-100 in PBS. Cell cycle phase distribution was analyzed after incubation with propidium iodide (PI, Sigma P4170, 0,04 mg/ml) using a flow cytofluorimeter Epics XL apparatus (Beckman Coulter). Parameters SS and FL-3 were acquired in a linear amplification scale, FS and Fl-2, in a log scale. Cell aggregates were gated out on the bi-parametric graph FL-3lin/Ratio. Apoptosis was determined as the proportion of cells exhibiting a DNA content lower than that of G1 cells after gating out cell debris on the bi-parametric graph FS/SS, using the WinMDI software.

### IF

Cells grown on coverslips were fixed as follows: (a) −20°C methanol, 6 minutes; or, for phospho-Aurora-B (Thr232) staining, (b) 3,7% PFA in PBS, 10 minutes at room temperature followed by 5 minutes in 0.1% TritonX-100 in PBS. Blocking and all antibody incubations were performed at room temperature in PBS/0.05% Tween 20/3% BSA. Cells were counterstained with 4,6-diamidino-2-phenylindole (DAPI, 0.1 μg/ml) and mounted using Vectashield (Vector Laboratories). Primary antibodies were: mouse anti-alpha-tubulin (1:2000, B-5-1-2, Sigma-Aldrich), mouse anti-Aurora-A (0.5 μg/ml, BD Transduction Laboratories), rabbit anti-phospho-Aurora-A (Thr288) (1:250, C39D8; Cell Signaling Technology), rabbit anti-pericentrin (2 μg/ml, ab4448; Abcam), rabbit anti-phospho-Aurora-B (Thr232) (1:50, Poly6361, BioLegend), mouse anti-phospho-Histone-H3 (Ser 10) (0.25 μg/ml, clone 3H10, Millipore), mouse anti-γ-tubulin (1:1000, GTU-88, Sigma-Aldrich), rabbit anti-lamin B1 (1 μg/ml, ab16048; Abcam), human anti-centromere (CREST; 1:20, Antibodies Incorporated). Samples were analyzed using a Nikon Eclipse 90i microscope equipped with a Qicam Fast 1394 CCD camera (QImaging). Image acquisition, deconvolution and Extended Depth of Focus on Z-serial optical sections were performed using Nis-Elements AR 4.2 (Nikon); images were further processed with Adobe Photoshop CS 8.0.

### Quantitative analysis of IF signals

Signals were measured using Nis Elements AR 4.2 (nd2 file format). Analysis of endogenous Aurora-A and Aurora-B activity in mitotic cells was performed as follows: a) p-Thr288-Aurora-A staining: average pixel intensity at spindle poles, corrected for external background; b) p-Thr232-Aurora-B staining: sum intensity at chromosomes, corrected for external background. Images for quantification of mitotic signals were Maximum Intensity Projections from z-stacks (0,6 μm, ranging over a 5-10 μm). Box-plots were generated using the web-tool BoxPlotR.

For measuring the angle between the centrosome-centrosome axis and the culture surface z-stacks serial images were used. The “arctan(xy/z)” formula for calculating the angle of a right triangle was applied with “xy” being the distance between centrosomes in xy in the maximum intensity projection, and “z” being the distance between centrosomes along the z-axis. A schematization is shown in Figure 6C. Values were statistically analyzed using the InStat3 software, using either (i) the unpaired t test (for Gaussian distributions), applying the Welch correction when required, or (ii) the Mann-Whitney test, when the populations did not follow a Gaussian distribution.

### Fluorescent in situ hybridization (FISH)

Cells grown on coverslips were fixed at room temperature with 3:1 methanol:acetic acid, air dried and stored at −20°C for at least one day. Before hybridization, coverslips were again incubated in 3:1 methanol:acetic acid, 1 hour at room temperature, then heated 2 hours at 65°C and dehydrated in 70%-90%-100% cold ethanol. Denaturation of probes in the hybridization mix (Fluorescein-labeled Chromosome 7 Satellite Probe, cat: PSAT0007-G; rhodamine-labeled Chromosome 11 Satellite Probe, cat: PSAT001-R; Hybridization Buffer QB007, all from Q-BIOgene) was performed at 96°C for 10 minutes. When the mix was applied on coverslips a co-denaturation step was performed (2 minutes, 72 °C), followed by the hybridization incubation, performed at 37°C, overnight. Coverslips were then washed in SSC, at 37°C and 60°C, and counterstained with DAPI (0.2 μg/ml) in SSC, 10 minutes at room temperature. Coverslips were mounted with Vectashield and analyzed with a Nikon Eclipse 90i microscope, using a 20x objective (Plan Fluor, 0,5 N.A.). Signals per nucleus were counted using the Object count function of Nis Elements AR 4.2 (nd2 file format), setting the parameters using the Spot detection function.

### Time-lapse recording

Cells seeded in 35 mm dishes (ibiTreat, cod. 81156, or glass bottom, cod. 81158, both from Ibidi) or 8-well micro-slides (ibiTreat, cod. 80826, Ibidi) were observed under an Eclipse Ti inverted microscope (Nikon), using 60x [Plan Apo, 1,4 N.A. Differential Interference Contrast (DIC), oil immersion] or 40x (Plan Fluor, 0,60 N.A. DIC) objectives (Nikon); during the whole observation cells were kept in a microscope stage incubator (Basic WJ, Okolab), at 37°C and 5% CO_2_. DIC images were acquired every 5 minutes over 24 hours using a DS-Qi1Mc camera and the NIS-Elements AR 3.22 software (Nikon). Image and movie processing were performed with NIS-Elements AR 4.2.

Imaging of the U2OS cell line stably expressing H2B-GFP and RFP-alpha-tubulin was performed with the 60x objective: images were acquired every 7 minutes in the 3 channels. z-stacks of the fluorescent channels were acquired every 1 μm over a range of 8 μm, attenuating the fluorescence lamp intensity to 1/32. Under these conditions, time-lapse acquisition, starting 6 hours after the treatment, was extended for 8 hours only to avoid phototoxic effects.

For high-throughput experiments, cells were seeded in 96-well plates. Images were acquired with a ScanR microscope (Olympus) using a 10x objective, an Olympus DBH1 camera and the ScanR acquisition software. Temperature and CO_2_ were kept constant by an incubation system on the microscope. Acquisition was performed every 30 minutes using Phase contrast and fluorescence imaging, for a total duration of 48-65 hours.

### Automated analysis of high-throughput data

Data from high-throughput experiments were analyzed by the CellCognition software (v 1.3.3-28, [28]). The software was used to segment the cells, extract features and classify them using a support vector machine. The classes defined were: interphases, prometaphases, metaphases, ana-telophases, multinucleated interphases, poliployd cells, dead cells; an additional class of debris or of cells with non homogeneous morphology which could not be included in any of the other categories was created and was then excluded from subsequent data elaboration to avoid artifacts. The classifier was trained with images from independent experiments with a training set comprising a minimum of 100 cells per class (with the exception of the polyploid class, of which only 33 examples were found). A confusion matrix and a classification test were used to assess the quality of classification before the analysis. The output data were further analyzed using Microsoft Excel; a single class for Mitoses was generated by pooling the prometaphase, metaphase and ana-telophase classes. Values for each well were pooled from 6 imaging fields; average values from 4 replicates within one experiment are shown in Figure 9. A similar trend was observed in 2 independent experiments (6 additional replicates).

### Immunoblotting

Mitotic cells collected by shake off were lysed in RIPA buffer (50 mM Tris-HCl pH 8.0, 150 mM NaCl, 1% NP40, 1 mM EGTA, 0.25% sodium deoxycholate) supplemented with protease and phosphatase inhibitors. Proteins were resolved by electrophoresis on 10% Laemmli gel and transferred on a nitrocellulose membrane (Protran BA83, Whatman) using a semi-dry system (BIO-RAD). 40 μg of extract per lane were loaded. Blocking and antibody incubations were performed at room temperature in PBS/0.1% Tween 20/5% low fat milk, or in PBS/0.1% Tween 20/5% BSA (for anti-phospho-Aurora-A hybridization). Antibodies were: mouse anti-Aurora-A (1 μg/ml; BD Transduction Laboratories), rabbit anti-phospho-Aurora-A (Thr288) (1:1000; C39D8; Cell Signaling Technology), goat anti-actin (0.5 μg/ml, I-19; SantaCruz Biotechnology). Signals were visualized by enhanced chemiluminescence detection (ECL plus, GE Healthcare, and Protein Detection System, GeneSpin).

## SUPPLEMENTARY FIGURES AND MOVIES










