# Supplementary material for: The Aurora-A inhibitor MLN8237 affects multiple mitotic processes and induces dose-dependent mitotic abnormalities and aneuploidy
Source: Oncotarget. 2014 Jul 9;5(15):6229–42. doi: 10.18632/oncotarget.2190 (PMC4171625; doi:10.18632/oncotarget.2190)
Supplement: Supplementary file 1 [file oncotarget-05-6229-s001.pdf]

The Aurora-A inhibitor MLN8237 affects multiple mitotic processes and induces dose-dependent mitotic abnormalities and aneuploidy

Supplementary Material

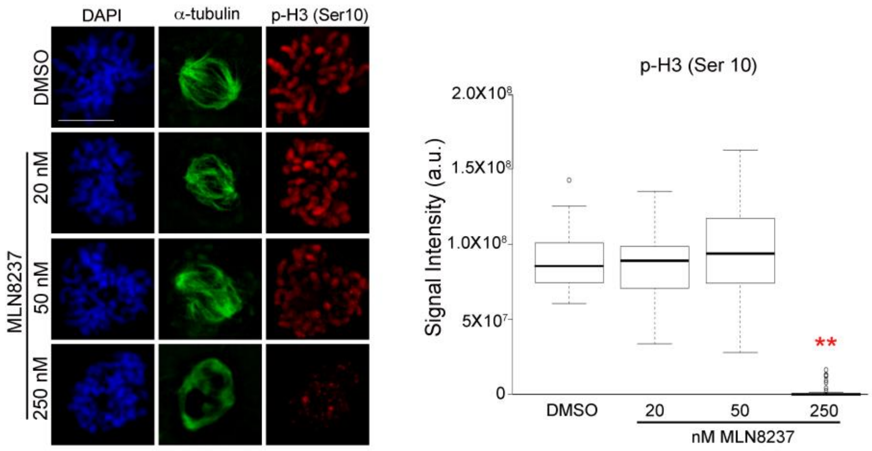

Supplementary Figure 1: Aurora-B inhibition after MLN8237 treatment assessed by phospho-Histone-H3 staining. Box-plots show phospho-H3 (Ser10) signal intensities (sum intensity over the whole chromosome area) in cultures treated with increasing MLN8237 concentrations (protocol in Figure 1): center lines show the medians; box limits indicate the 25th and 75th percentiles as determined by R software; whiskers extend 1.5 times the interquartile range from the 25th and 75th percentiles, outliers are represented by dots. 50 mitoses per condition from 2-3 experiments were measured. **: $p < 0.0001$, Mann-Whitney test (InStat3 software). Representative immunofluorescence images are shown on the left. Scale bar: 10 μm.

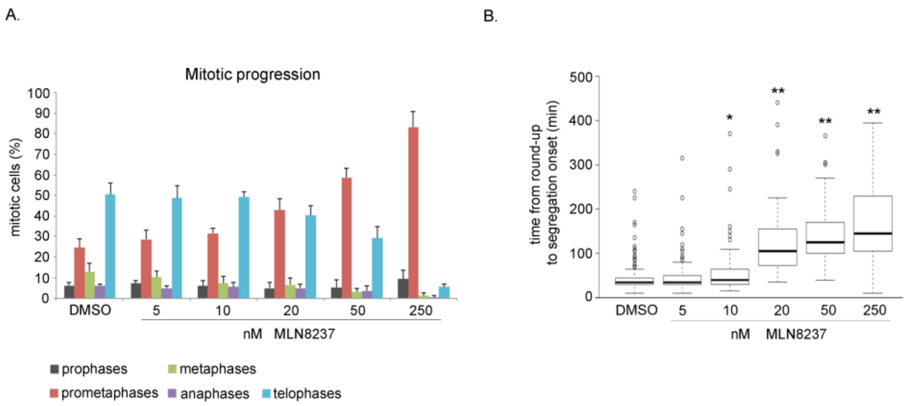

Supplementary Figure 2: Prometaphase is prolonged following Aurora-A inhibition. A. Histograms represent the percentage of cells in different mitotic phases under the indicated conditions (at least 250 scored mitoses per condition, 2 experiments). Error bars denote s.d. B. Time from round-up to the onset of segregation (or abnormal mitotic exit at high MLN8237 concentrations) is shown in the box-plots (center lines show the medians; box limits indicate the 25th and 75th percentiles as determined by R software; whiskers extend 1.5 times the interquartile range from the 25th and 75th percentiles, outliers are represented by dots). 60-200 mitoses per condition from 2-3 experiments were recorded. * p=0,0004; **: $p < 0.0001$, Mann-Whitney test (InStat3 software).

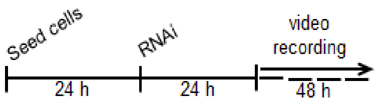

	control	Aurk-Ai	Aurk-Bi	Aurk-A+Bi
Division attempt and re-adhesion (%)	0,00	0,00	6,15*	6,98*
Re-adhesion without segregation (%)	0,00	0,00	**15,38****	**41,86****
	n=84	n=50	n=65	n=43

Supplementary Figure 3: Cell division failure in Aurora-A+Aurora-B interfered mitoses. The protocol for RNA interference-mediated inactivation of Aurora-A and Aurora-B, either singularly (Aurk-Ai; Aurk-Bi) or in combination (Aurk-A+Bi) is shown on top. Trasfection was performed as described, using the neutral GL2 oligonucleotide as control; siRNA oligonucleotides were used 80 nM for Aurora-A (Asteriti et al., 2011) and 50 nM for Aurora-B (Ditchfield et al., 2003). Time-lapse recording started 24 hours after transfection and spanned the next 48 hours. Mitoses that re-adhered in one single cell either without chromosome segregation, or after a failed segregation attempt, were scored; percentages are shown in the table below (* $p<0,05$; ** $p<0,001$; $\chi 2$ test).

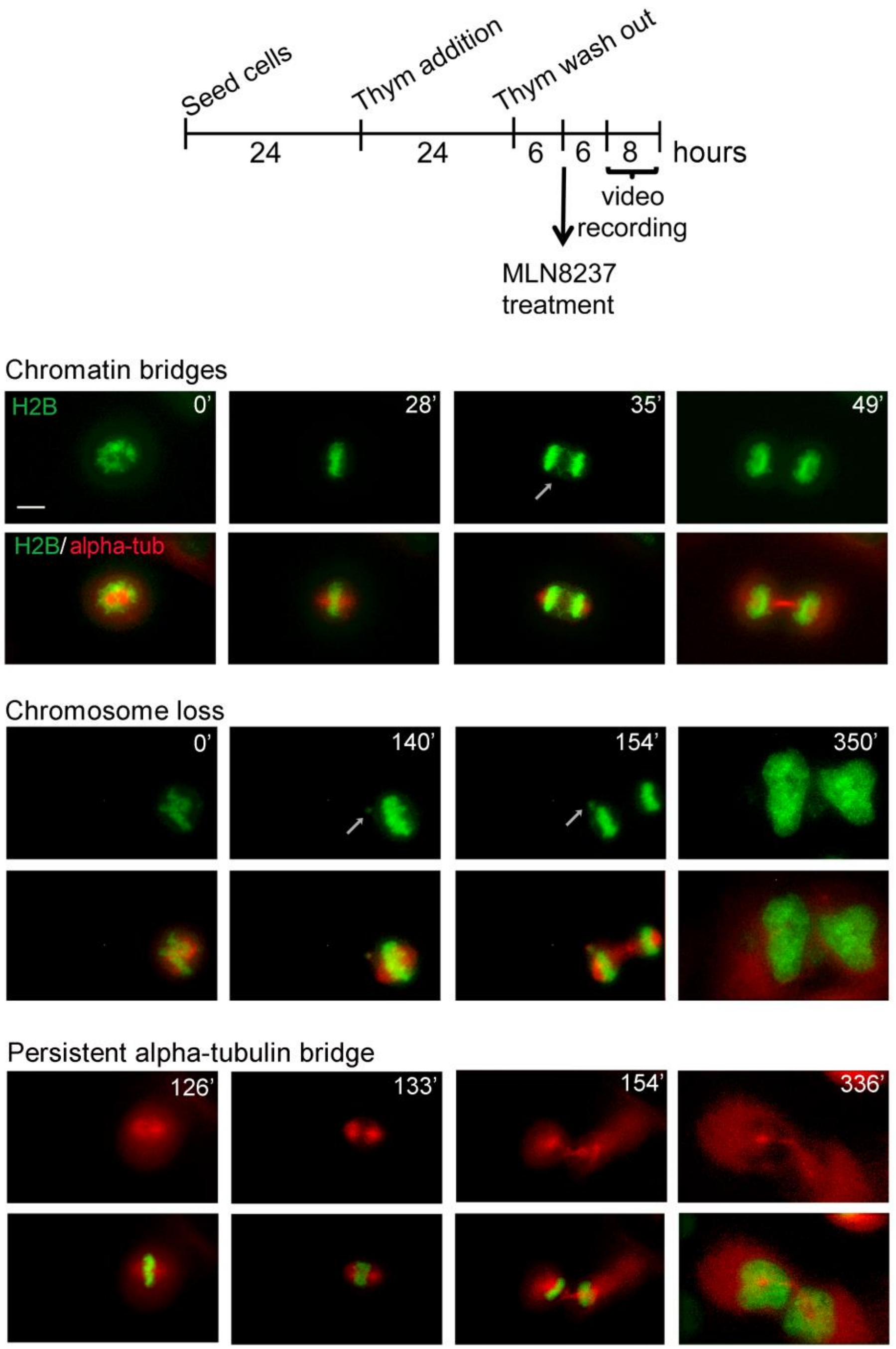

Supplementary Figure 4: Dynamic defects in late mitoses in MLN8237-treated cultures. A schematization of the protocol for recording MLN8237-treated mitoses in U2OS cells with fluorescently labeled H2B and alpha-tubulin is shown on top (Thym: thymidine). Single photograms (Maximum Intensity Projections) from videos are shown below, representative of defects arising in ana-telophase. Minutes from prometaphase onset, as assessed by the H2B signal, are indicated. Arrows in upper and middle panels indicate mis-segregating chromosomes. Scale Bar: 10 µm.

Supplementary Movie S1: U2OS control (DMSO-treated) mitoses recorded by time-lapse imaging (5 minutes intervals) using a 40x Differential Interference Contrast objective.

Supplementary Movie S2: U2OS MLN8237-treated mitosis displaying the multipolar phenotype, recorded by time-lapse imaging (5 minutes intervals) using a 40x Differential Interference Contrast objective.

Supplementary Movie S3: U2OS MLN8237-treated cells exiting mitosis without chromosome segregation, recorded by time-lapse imaging (5 minutes intervals) using a 40x Differential Interference Contrast objective.

Supplementary Movie S4: U2OS MLN8237-treated mitosis displaying the mis-oriented division phenotype, recorded by time-lapse imaging (5 minutes intervals) using a 40x Differential Interference Contrast objective.
